# Microbial Community Analysis of a Coastal Salt Marsh Affected by the *Deepwater Horizon* Oil Spill

**DOI:** 10.1371/journal.pone.0041305

**Published:** 2012-07-18

**Authors:** Melanie J. Beazley, Robert J. Martinez, Suja Rajan, Jessica Powell, Yvette M. Piceno, Lauren M. Tom, Gary L. Andersen, Terry C. Hazen, Joy D. Van Nostrand, Jizhong Zhou, Behzad Mortazavi, Patricia A. Sobecky

**Affiliations:** 1 Department of Biological Sciences, University of Alabama, Tuscaloosa, Alabama, United States of America; 2 Lawrence Berkeley National Laboratory, Berkeley, California, United States of America; 3 Department of Microbiology, University of Tennessee, Knoxville, Tennessee, United States of America; 4 Institute for Environmental Genomics, University of Oklahoma, Norman, Oklahoma, United States of America; 5 Dauphin Island Sea Lab, Dauphin Island, Alabama, United States of America; Auburn University, United States of America

## Abstract

Coastal salt marshes are highly sensitive wetland ecosystems that can sustain long-term impacts from anthropogenic events such as oil spills. In this study, we examined the microbial communities of a Gulf of Mexico coastal salt marsh during and after the influx of petroleum hydrocarbons following the *Deepwater Horizon* oil spill. Total hydrocarbon concentrations in salt marsh sediments were highest in June and July 2010 and decreased in September 2010. Coupled PhyloChip and GeoChip microarray analyses demonstrated that the microbial community structure and function of the extant salt marsh hydrocarbon-degrading microbial populations changed significantly during the study. The relative richness and abundance of phyla containing previously described hydrocarbon-degrading bacteria (*Proteobacteria, Bacteroidetes,* and *Actinobacteria*) increased in hydrocarbon-contaminated sediments and then decreased once hydrocarbons were below detection. *Firmicutes,* however, continued to increase in relative richness and abundance after hydrocarbon concentrations were below detection. Functional genes involved in hydrocarbon degradation were enriched in hydrocarbon-contaminated sediments then declined significantly (p<0.05) once hydrocarbon concentrations decreased. A greater decrease in hydrocarbon concentrations among marsh grass sediments compared to inlet sediments (lacking marsh grass) suggests that the marsh rhizosphere microbial communities could also be contributing to hydrocarbon degradation. The results of this study provide a comprehensive view of microbial community structural and functional dynamics within perturbed salt marsh ecosystems.

## Introduction

The explosion and sinking of the *Deepwater Horizon* drilling rig on April 20, 2010 released an estimated 4.9 million barrels of crude oil [1], [2], [3] and an estimated 1.7×10^11^ g of volatile hydrocarbons [4] into the northern Gulf of Mexico (GoM). Crude oil reached the coast of Alabama by June 2010 and after approximately one month at sea the oil that impacted coastal areas was weathered and presented in the form of oil sheens and tar balls/mats [5], [6].

Coastal wetlands are among the most productive habitats in the world that act as nurseries for fisheries, sinks for anthropogenic contaminants, and provide protection from shoreline erosion [7]. Wetland environments are highly sensitive to oil contamination due to low tidal wave energy and the presence of marsh grasses that allow pollutants to remain sequestered in coastal sediments for years [8], [9]. Traditional mechanical remediation processes, such as low-pressure flushing and vacuum/pumping, may adversely affect marsh environments or irreparably damage these sensitive ecosystems [10], [11]. Two coastal marshes equally affected by heavy oiling from the *Amoco Cadiz* in 1978 were compared after one site received aggressive cleaning and the other was left untouched. Fifteen years later the cleaned marsh showed ∼30% decrease in vegetative area compared to a 21% increase in the size of the marsh that did not receive cleanup [12]. Thus, in many cases natural attenuation through microbial oil biodegradation processes is a preferred course of action in contaminated salt marshes to limit or prevent long-term habitat loss.

**Figure 1:**
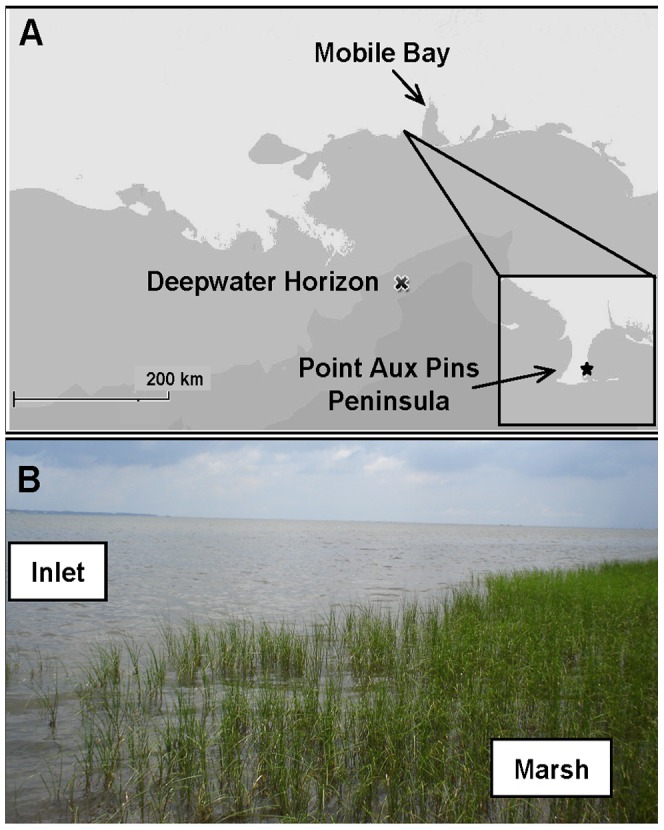
Study site. (A) Map of Point Aux Pins peninsula. Study site indicated by box in inset. (B) Sediment samples collected ∼25 m from shore outside the marsh stands (designated “Inlet”) and 2–4 m from shore within the marsh stands (designated “Marsh”).

A number of environmental factors can enhance or limit natural attenuation or *in situ* bioremediation of oil, including temperature, salinity, nutrient availability, oxygen, physical state of the oil, and the natural microbial community [13]. Biodegradation of hydrocarbons is enhanced at higher temperatures (during summer months) when microbial activity is increased and oil viscosity is lower when compared to winter months. Tar balls and/or mousse are less viscous at higher temperatures and have more surface area available for physical breakdown and/or microbial degradation within sediments [14]. The most rapid rates of microbial hydrocarbon degradation occur under aerobic conditions when compared to anaerobic hydrocarbon degradation [15], [16]. Inorganic nutrients (e.g. nitrogen, phosphorus, and iron) essential for microbial hydrocarbon degradation are replete in many marsh systems (due to land run-off). Another critical factor affecting the bioremediation of oil is the structure of the natural microbial community. Within GoM coastal ecosystems, microbial communities have adapted to hydrocarbon exposure as a result of chronic release from natural hydrocarbon seeps [17], [18], [19], [20], [21]. Thus, these GoM microbial communities represent a unique contrast to those present within coastal ecosystems that do not have exposure to hydrocarbon release.

**Table 1:** Point Aux Pins salt marsh inlet water column physical and chemical parameters.

	June 8, 2010	July 2, 2010	September 10, 2010
**Temperature (°C)**	34.3	30.2	34.4
**Salinity (ppt)**	17.0	15.1	22.4
**Total Dissolved Solids (g L^−1^)**	18.1	15.5	23.3
**pH**	8.48	7.49	8.39
**Dissolved Oxygen (%)**	141.2	84.9	110.4

Dissolved oxygen concentrations reported relative to 100% air saturation.

There is a paucity of information regarding the *in situ* structure and function of salt marsh microbial communities in coastal GoM environments impacted by oil contamination. To date, studies that examine the microbial response to the *Deepwater Horizon* oil spill have described beach [22], [23] and open ocean [2], [24], [25], [26], [27] environments. To the best of our knowledge, salt marsh microbial community structure and function have not been characterized by high-density PhyloChip and GeoChip microarray analyses. These high throughput culture-independent analyses have been shown to identify a greater microbial diversity in environmental samples compared to traditional sequencing of clone libraries [28]. Therefore, the goal of this study was to employ microarray-based methods to characterize the changes to the microbial community within a coastal salt marsh during and after oiling from the *Deepwater Horizon*.

**Table 2:** Total petroleum hydrocarbons (TPH) detected in one or replicate samples.

Sample Location	Date collected	Sediment Depth (cm)	Total Petroleum Hydrocarbons (mg kg^−1^)
**Tar balls**	July 2010	–	20,300
**Inlet**	June 2010	0–2	BD, BD, BD
**Inlet**	June 2010	8–10	BD, 36, 145
**Inlet**	July 2010	0–2	BD, BD, 164
**Inlet**	July 2010	8–10	BD, 32, 64
**Inlet**	Sept 2010	0–2	48, 49, 56
**Inlet**	Sept 2010	8–10	BD, BD
**Marsh**	June 2010	0–2	47, 54, 189
**Marsh**	June 2010	8–10	34, 38, 175
**Marsh**	July 2010	0–2	53, 66, 163
**Marsh**	July 2010	8–10	BD, 31, 33
**Marsh**	Sept 2010	0–2	BD, BD, BD
**Marsh**	Sept 2010	8–10	BD, BD, BD

* BD indicates total petroleum hydrocarbons were below limit of detection.

**Figure 2:**
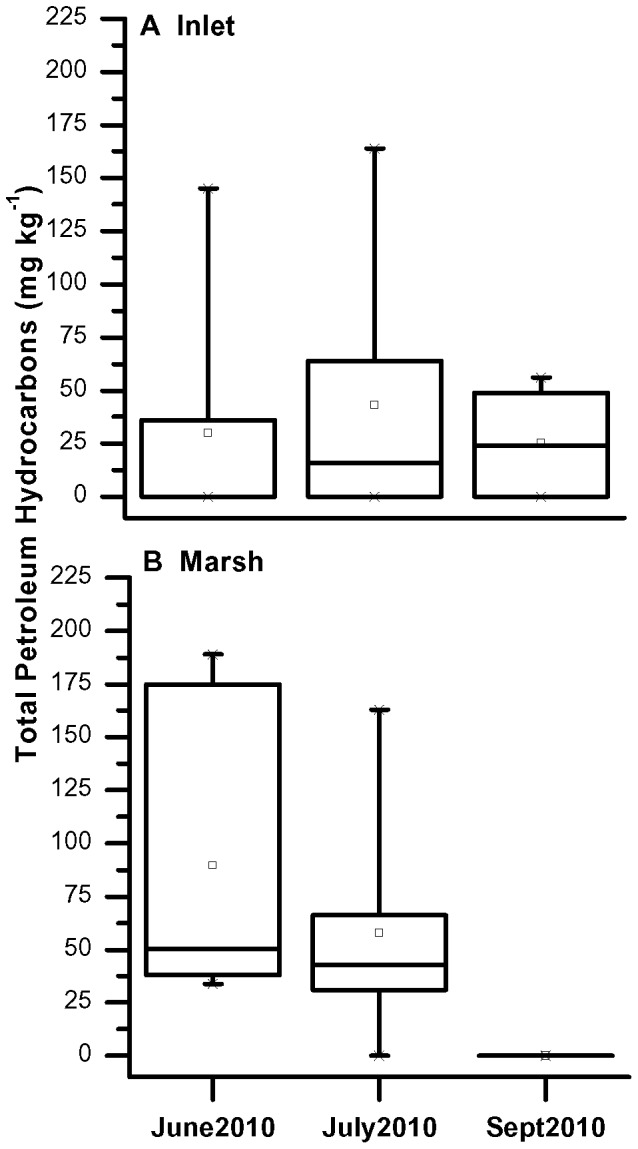
Boxplots of TPH concentrations in (a) Inlet and (b) Marsh surface and subsurface sediments. Each box represents the concentration range of 50% of the observations. Lines within the boxes represent the median values, open squares are the averages, and the whisker lines connect to outliers.

## Materials and Methods

### Study Site

The salt marsh study site was located on the eastern side of the Point Aux Pins peninsula, southwest of Bayou La Batre, Alabama (30°22′ N; 88°18′ W) (Figure 1a). The marsh is dominated by *Juncus roemerianus* and bordered by *Spartina alterniflora* along the outer edge. The rush stands extend approximately 10 m out from the shoreline. Water column depth during summer months was approximately 1 m. Tides are diurnal (daily) and of moderate energy and amplitude (∼0.38 m) [29]. No specific permits or permissions were required for the described field studies at this location, which is not privately-owned or protected, and the field studies did not involve endangered or protected species.

**Figure 3:**
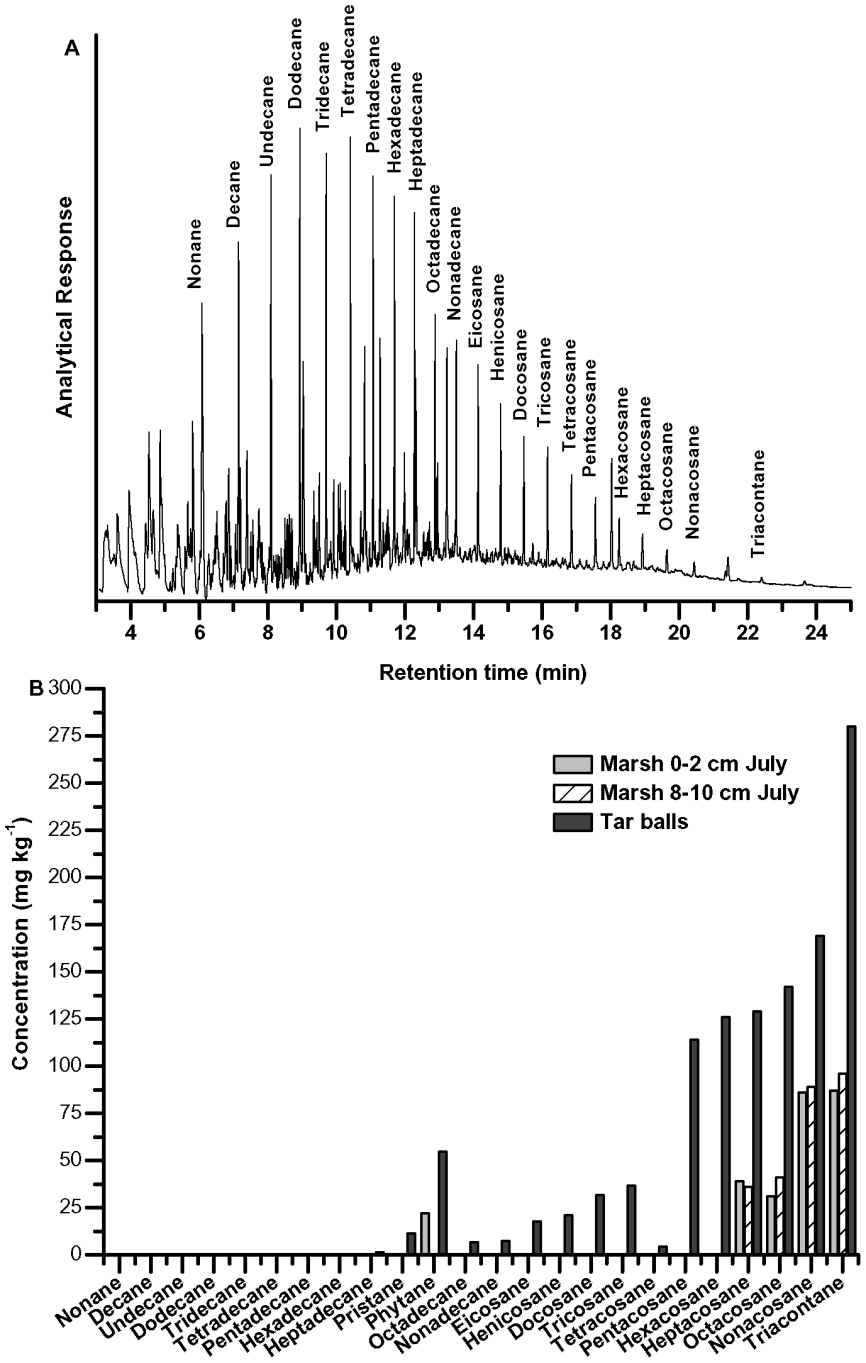
GCMS analysis of MC252 oil and oil-contaminated sediment. (A) GCMS chromatogram of 10 ppm Macondo oil (MC252); (B) concentrations of *n*-alkanes in tar balls and sediment from July Marsh samples as determined by GCMS.

### Sample Collection and Processing

Triplicate sediment cores (30 cm; 7.3 cm I.D.) were collected within the marsh stands (designated “Marsh”) approximately 2–4 m from shore and outside the stands approximately 25 m from the shoreline (designated “Inlet”) (Figure 1b). Sediment cores were collected from the same location within the salt marsh during each sampling period. All samples were stored at 4°C and −80°C prior to processing. Sampling occurred on June 8, July 2, and September 10, 2010. Tar balls and tar mats mixed with wrack (organic debris) were collected in triplicate along the marsh fringe and shoreline on July 2, 2010. Sediment cores were aseptically extruded in 2 cm sections with the 0–2 cm and 8–10 cm sections used for analysis. Inlet water column temperature, salinity, dissolved oxygen, total dissolved solids, and pH were measured with a handheld YSI 556 multiprobe meter (YSI Incorporated, Yellow Springs, Ohio USA).

**Table 3:** Number of bacterial OTUs detected by PhyloChip in Inlet and Marsh sediments for June, July, and September 2010.

	Inlet 0–2 cm			Inlet 8–10 cm			Marsh 0–2 cm			Marsh 8–10 cm		
Phylum	June	July	September	June	July	September	June	July	September	June	July	September
*Acidobacteria*	60	201	161	88	98	105	60	183	68	81	173	114
*Actinobacteria*	231	734	603	284	329	410	229	770	452	265	657	444
*Bacteroidetes*	185	488	555	179	204	134	139	689	75	158	445	154
*Chlorobi*	5	15	17	11	12	12	3	17	9	8	19	12
*Chloroflexi*	46	139	99	68	71	79	61	158	73	73	169	100
*Cyanobacteria*	51	158	122	70	75	52	47	154	36	39	130	76
*Firmicutes*	150	830	1262	276	312	975	117	1050	1560	157	1013	787
* Bacilli*	52	246	514	88	118	461	50	298	714	51	299	330
* Clostridia*	98	584	748	188	194	514	67	752	846	106	714	457
*Fusobacteria*	10	27	16	14	8	5	13	32	2	2	29	3
*Gemmatimonadetes*	26	66	62	33	38	37	15	58	29	29	63	45
*Nitrospirae*	5	18	14	11	9	9	4	20	3	5	19	12
*Planctomycetes*	76	166	117	90	85	79	74	157	45	77	147	97
*Proteobacteria*	1140	3196	3843	1441	1560	1719	713	3625	1019	1084	2439	1684
* Alphaproteobacteria*	151	720	820	154	138	332	113	903	206	108	457	312
* Betaproteobacteria*	145	411	487	161	182	278	109	452	249	124	320	261
* Deltaproteobacteria*	164	560	438	244	270	278	159	595	172	209	567	318
* Epsilonproteobacteria*	36	54	107	88	98	89	44	90	27	84	104	90
* Gammaproteobacteria*	642	1446	1983	792	869	740	288	1577	364	558	986	699
Unclassified	0	1	4	0	1	0	0	3	0	0	1	1
* Zetaproteobacteria*	2	4	4	2	2	2	0	5	1	1	4	3
SAR406	4	26	20	12	13	17	5	25	15	10	24	19
*Spirochaetes*	16	67	64	31	36	44	19	65	35	20	73	43
*Synergistetes*	3	10	11	4	6	6	3	11	6	2	11	7
*Tenericutes*	8	59	68	18	19	53	12	81	68	17	72	39
*Verrucomicrobia*	36	142	99	35	37	37	34	138	22	26	90	41
WS3	15	36	28	23	23	25	15	33	17	21	34	23
Other/Unclassified	66	199	178	109	117	134	62	197	104	98	228	159
**Total**	**2133**	**6577**	**7339**	**2797**	**3052**	**3932**	**1625**	**7463**	**3638**	**2172**	**5835**	**3859**

### DNA Extraction

Genomic DNA was extracted in triplicate from 1 g sediment with an MP Biomedicals FastDNA spin kit for soils (MP Biomedicals, Solon, OH) according to the manufacturer’s protocol. DNA concentrations were measured via absorption at 260 nm using a NanoDrop ND-1000 (Thermo Scientific, Beverly, MA).

**Table 4:** Relative abundance (%) of families of *Actinobacteria*, *Bacteroidetes*, and *Firmicutes* known to contain hydrocarbon-degrading species.

				Relative Abundance (%)					
				Inlet			Marsh		
Phylum	Class	Order	Family	June	July	Sept	June	July	Sept
*Actinobacteria*	*Actinobacteria*	*Actinomycetales*	*Actinomycetaceae*	0	24.7	12.6	16.7	35.8	10.2
			*Brevibacteriaceae*	6.1	19.0	18.0	18.1	23.6	15.2
			*Corynebacteriaceae*	12.2	16.9	17.0	14.8	20.6	18.6
			*Dietziaceae*	0.0	49.9	20.6	0.0	29.5	0.0
			*Gordoniaceae*	16.2	16.0	18.4	11.9	23.3	14.2
			*Microbacteriaceae*	7.6	22.2	18.7	12.2	25.1	14.2
			*Micrococcaceae*	8.9	19.3	18.6	12.3	27.8	13.2
			*Mycobacteriaceae*	7.0	19.3	16.0	11.9	31.8	13.9
			*Nocardiaceae*	8.5	18.2	15.7	15.6	26.4	15.7
			*Nocardioidaceae*	5.0	24.7	16.0	9.0	36.1	9.1
			*Streptomycetaceae*	6.8	24.0	16.8	14.7	24.2	13.4
*Bacteroidetes*	*Flavobacteriia*	*Flavobacteriales*	*Flavobacteriaceae*	12.2	19.4	20.8	15.5	25.6	6.4
	*Sphingobacteria*	*Sphingobacteriales*	*Flexibacteraceae*	9.0	21.8	15.3	17.0	27.7	9.1
*Firmicutes*	*Bacilli*	*Bacillales*	*Bacillaceae*	4.6	9.8	26.1	6.5	13.6	39.5
			*Paenibacillaceae*	2.5	9.3	37.8	6.3	14.1	30.0
			*Planococcaceae*	3.8	16.6	28.8	8.2	18.4	24.2
			*Staphylococcaceae*	6.3	15.2	23.3	9.9	16.8	28.5
		*Lactobacillales*	*Lactobacillaceae*	6.0	18.1	19.3	10.1	23.6	22.9
	*Clostridia*	*Clostridiales*	*Clostridiaceae*	4.5	12.4	20.4	7.7	19.7	35.1
			*Peptococcaceae*	9.8	15.8	18.6	11.9	21.5	22.3
**Overall relative abundance of entire bacterial community**				**10.2**	**19.0**	**19.8**	**13.2**	**23.0**	**14.7**

**Table 5:** Relative abundance (%) of families of *Proteobacteria* known to contain hydrocarbon-degrading species.

			Relative Abundance (%)					
			Inlet			Marsh		
Class	Order	Family	June	July	Sept	June	July	Sept
*α-proteobacteria*	*Caulobacterales*	*Caulobacteraceae*	5.2	16.9	27.4	9.6	28.3	12.7
	*Rhizobiales*	*Beijerinckiaceae*	4.2	20.5	29.4	8.5	27.0	10.5
		*Brucellaceae*	6.4	13.4	33.2	10.8	25.8	10.3
		*Hyphomicrobiaceae*	7.7	18.6	18.5	12.6	30.8	11.8
		*Rhizobiaceae*	4.1	16.8	28.5	9.1	31.4	10.0
		*Xanthobacteraceae*	11.6	15.7	23.1	11.5	33.5	4.7
	*Rhodospirillales*	*Acetobacteraceae*	6.0	15.4	21.3	10.2	22.6	24.5
		*Rhodospirillaceae*	8.0	19.6	20.4	12.6	26.5	12.9
	*Sphingomonadales*	*Erythrobacteraceae*	4.2	20.2	25.5	9.0	32.9	8.2
		*Sphingomonadaceae*	7.1	18.7	23.0	11.9	26.3	13.0
*ß-proteobacteria*	*Burkholderiales*	*Alcaligenaceae*	8.8	18.3	22.9	9.9	20.8	19.3
		*Burkholderiaceae*	6.7	13.6	25.9	12.2	24.6	17.0
		*Comamonadaceae*	10.2	17.6	19.3	13.9	22.5	16.5
	*Rhodocyclales*	*Rhodocyclaceae*	9.4	14.4	25.2	12.0	18.0	21.1
*δ-proteobacteria*	*Desulfuromonadales*	*Geobacteraceae*	8.2	21.5	17.3	12.8	28.7	11.5
	*Syntrophobacterales*	*Desulfobacteraceae*	13.4	20.2	15.3	15.7	24.5	10.8
*γ-proteobacteria*	*Aeromonadales*	*Aeromonadaceae*	15.0	40.6	20.3	8.4	9.8	5.9
	*Alteromonadales*	*Alteromonadaceae*	14.5	14.8	29.6	20.0	13.0	8.1
		*Colwelliaceae*	22.1	15.6	24.7	15.9	14.6	7.0
		*Pseudoalteromonadaceae*	19.2	22.3	20.1	13.1	18.0	7.4
		*Shewanellaceae*	26.4	27.2	17.8	17.3	8.4	2.9
	*Enterobacteriales*	*Enterobacteriaceae*	13.6	22.3	21.0	14.5	18.0	10.6
	*Oceanospirillales*	*Alcanivoracaceae*	10.1	16.3	26.9	15.7	16.7	14.2
		*Alteromonadaceae*	8.9	19.4	20.8	14.1	26.0	10.7
		*Halomonadaceae*	10.9	21.1	20.7	13.0	23.7	10.5
		*Oceanospirillaceae*	22.9	17.8	23.3	13.1	13.2	9.7
		*Oleiphilaceae*	11.7	16.1	28.8	13.6	15.8	13.9
	*Pasteurellales*	*Pasteurellaceae*	9.8	19.6	24.9	16.8	21.1	7.7
	*Pseudomonadales*	*Moraxellaceae*	9.4	17.0	23.9	12.3	21.9	15.6
		*Pseudomonadaceae*	15.3	21.4	26.7	12.0	13.7	10.9
	*Thiotrichales*	*Piscirickettsiaceae*	9.2	13.5	28.1	13.9	17.8	17.6
		*Thiotrichaceae*	9.2	17.9	20.7	16.4	22.5	13.3
	*Vibrionales*	*Vibrionaceae*	15.6	19.5	17.3	14.6	26.7	6.3
	*Xanthomonadales*	*Xanthomonadaceae*	8.9	12.4	33.2	10.5	15.8	19.1
**Overall relative abundance of entire bacterial community**			**10.2**	**19.0**	**19.8**	**13.2**	**23.0**	**14.7**

### PCR Amplification of 16S rRNA Genes and Microarray Analysis (PhyloChip and GeoChip)

Polymerase chain reaction (PCR) of 16S rRNA genes was performed with universal *Bacteria* (27F and 1492R) primers. Reagents for all PCR reactions and thermocycling conditions for bacterial 16S rRNA genes were performed as previously described [27], except that bacterial product was amplified in 25 cycles (instead of 30 cycles). Bacterial 16S rRNA gene diversity was assessed for June, July, and September 2010 sediments using the G3 PhyloChip, an Affymetrix platform microarray. The G3 PhyloChip contains over 1 million probes and can identify up to nearly 60,000 operational taxonomic units (OTUs). OTUs were scored as “present” when at least two of the three replicate core samples contained the OTU. Microarray sample preparation, hybridization, and scaling were performed as previously described [27]. Microbial functional gene analysis was conducted using the GeoChip 2.0 for June and July 2010 sediment samples. The GeoChip 2.0 microarray contains probes for >10,000 genes in >150 functional groups [30], [31]. Microarray sample preparation, hybridization, and normalization were performed on triplicate samples as previously described [30], [32]. Data analyses were performed with Primer-E software (version 6.1.13, Plymouth Marine Laboratory). Non-metric multi-dimensional scaling (nMDS) ordination of the bacterial community data was generated from a Bray Curtis resemblance matrix based on the mean relative abundance of taxa identified by PhyloChip in triplicate cores. Student’s *t*-test and analysis of similarities (ANOSIM) were performed for sample comparisons.

**Figure 4:**
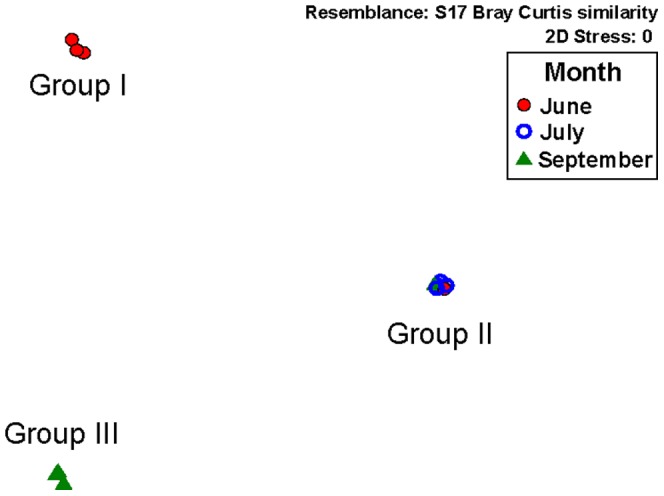
Non-metric multi-dimensional scaling (nMDS) ordination of the bacterial community in salt marsh sediments. Resemblance matrix generated using Bray Curtis similarity and based on the relative abundance of taxa identified by PhyloChip analysis.

### Hydrocarbon Analysis

Total petroleum hydrocarbons (TPH) were extracted from sediment (30–60 g) collected in June, July, and September according to the EPA SM5520 method and analyzed by Fourier transform infrared spectroscopy (FTIR). TPH detection limit was 20 mg kg^−1^ for sediment samples. Tar balls and selected sediment samples (30–60 g) were extracted for *n*-alkanes with methylene chloride and analyzed by gas chromatography-mass spectrometry (GCMS). The crude oil calibration curve was generated using a sample of Macondo oil (MC252) collected from the wellhead (provided by T. Hazen courtesy of BP). All hydrocarbon measurements were performed by Envirochem Environmental Laboratories, Mobile, AL.

**Figure 5:**
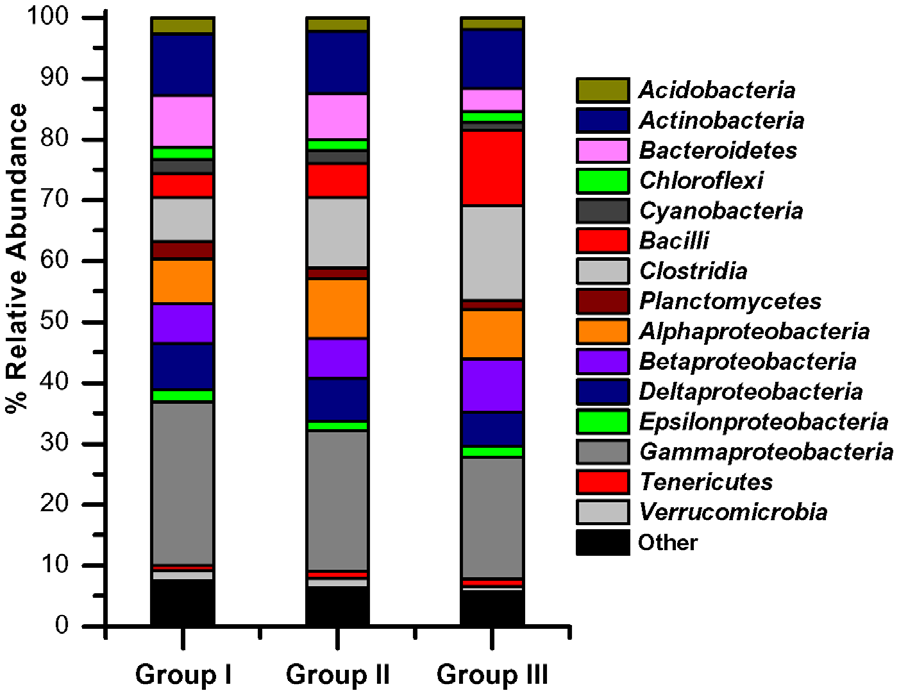
Relative abundance of phyla in salt marsh sediments. Groups I, II, and III determined from nMDS analysis.

## Results

### Site Water Parameters

Water quality parameters measured during the June, July, and September 2010 sampling periods are summarized in Table 1. Water temperature averaged 33°C over the four month time period (∼1 m depth). Salinity ranged between 15 and 17 ppt in June and July and increased to 22 ppt in September. Dissolved oxygen saturation (DO) was highest (141%) in June and lowest in July (84%). DO increased to 110% in September 2010 (Table 1). Turbidity correlated with salinity and pH ranged between 7.5 and 8.5.

**Figure 6:**
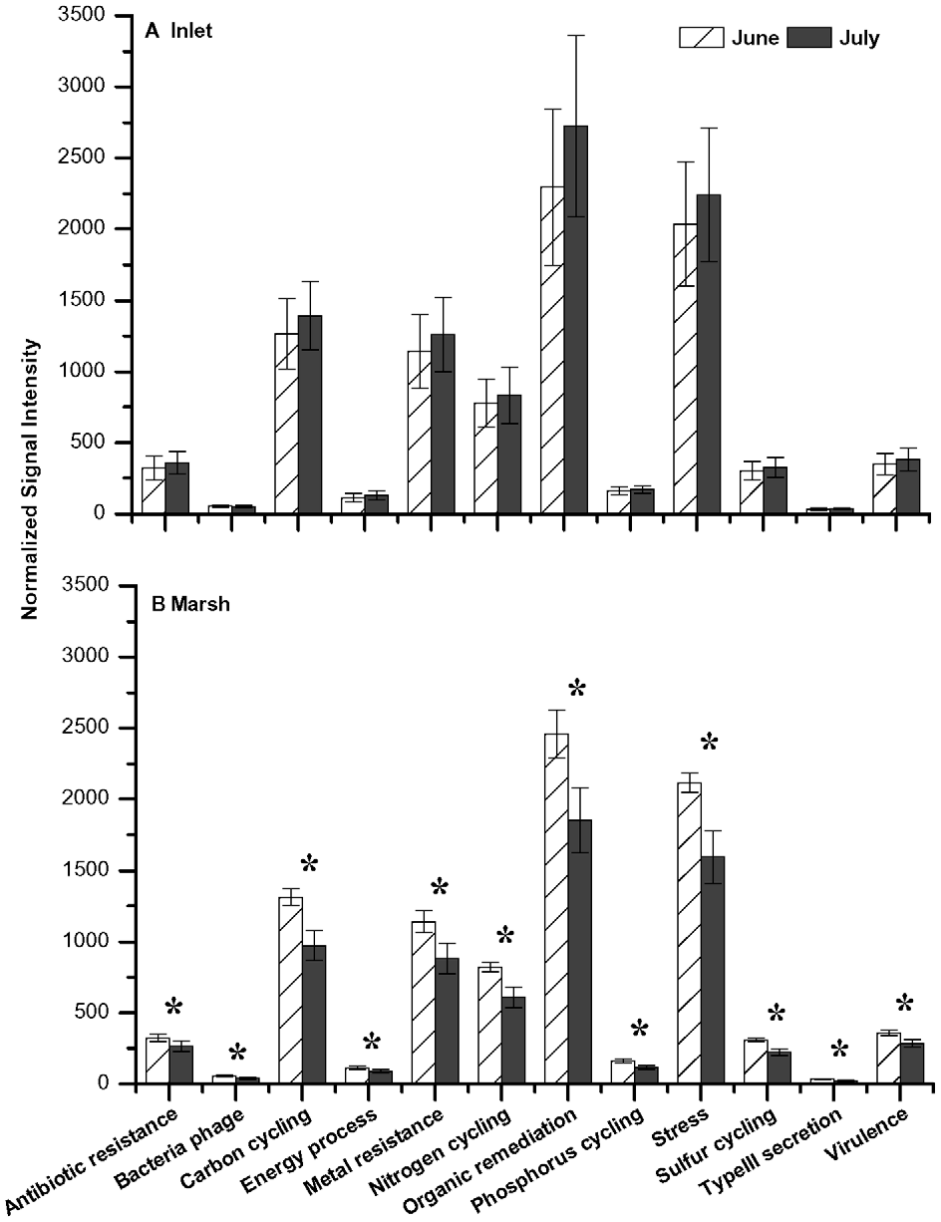
Functional gene distribution in Inlet and Marsh sediments. Gene relative abundance determined by GeoChip for samples compared between June and July 2010. Categories with significant changes indicated by *for p<0.05.

### Hydrocarbons

Hydrocarbons were analytically detected in salt marsh sediments in June 2010. During sampling in July 2010, oil was present as a light sheen or mousse floating on the water and tar mats were observed mixed with wrack (organic debris) on the rush stands, marsh fringe, and shoreline. By early September 2010 oil was no longer visible in the water or along the shoreline of the marsh. TPH ranged from below detection (BD) to 189 mg kg^−1^ in the sediments and was highest at 20,300 mg kg^−1^ in tar balls (Table 2). TPH was distributed heterogeneously in the Inlet site samples as demonstrated by the wide range of values measured in replicate cores. TPH concentrations in June Inlet replicate cores were BD at the sediment-water interface within the 0–2 cm sections, however deeper in the core (8–10 cm) TPH varied from BD to 145 mg kg^−1^ suggesting that oil was randomly dispersed throughout the Inlet. July Inlet cores demonstrated similar heterogeneity with TPH ranging from BD to 164 mg kg^−1^ at the surface and BD to 64 mg kg^−1^ in subsurface sections. TPH was detected in surficial Inlet sediments in September (∼50 mg kg^−1^), but was BD at 8–10 cm depth. TPH in the June Marsh samples were more evenly distributed than in the Inlet samples, with TPH concentrations above detection in all replicate cores at both the surface and in the subsurface. TPH measurements varied within the replicates from 34 to 189 mg kg^−1^. July Marsh cores demonstrated a similar trend as June cores at the surface, with TPH ranging from 53 to 163 mg kg^−1^, but TPH decreased in subsurface sections ranging from BD to 33 mg kg^−1^. By September 2010 TPH was BD in all Marsh samples.

**Figure 7:**
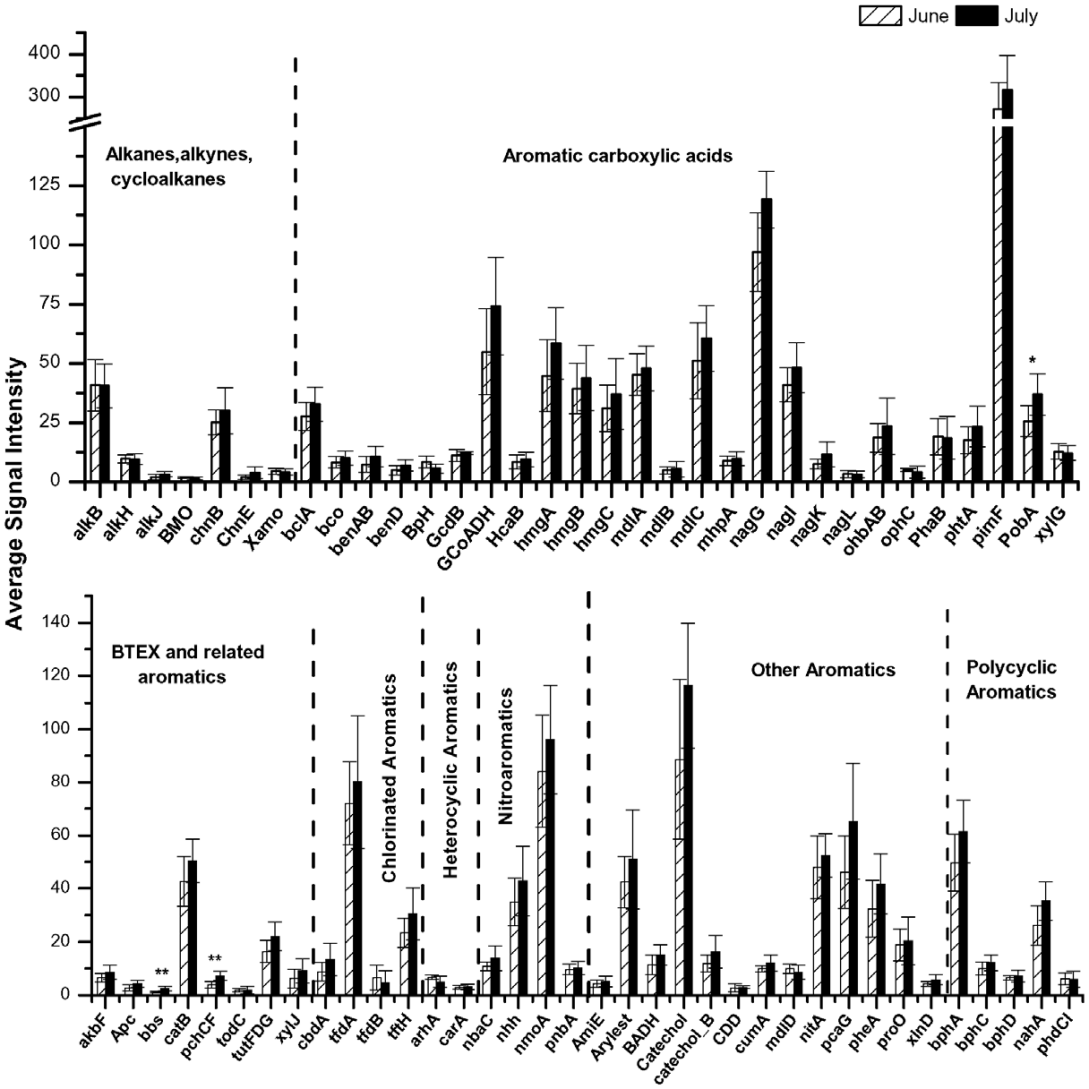
Hydrocarbon degradation genes in Inlet sediments. Average abundances of hydrocarbon degradation genes in Inlet sediments compared between June and July. Significant changes indicated by *for p<0.05 and **for p<0.01.

The range of TPH concentrations in Inlet samples was relatively constant in June, July, and September with the majority (50%) of samples ranging from BD to approximately 50 mg kg^−1^ (Figure 2). In contrast, the range of TPH in Marsh samples was distinctly different between the months with the majority of samples in June having TPH ranging from 50 to 175 mg kg^−1^. By July, the average TPH concentration decreased between 25 to 55 mg kg^−1^ and was below detection by September.

**Figure 8:**
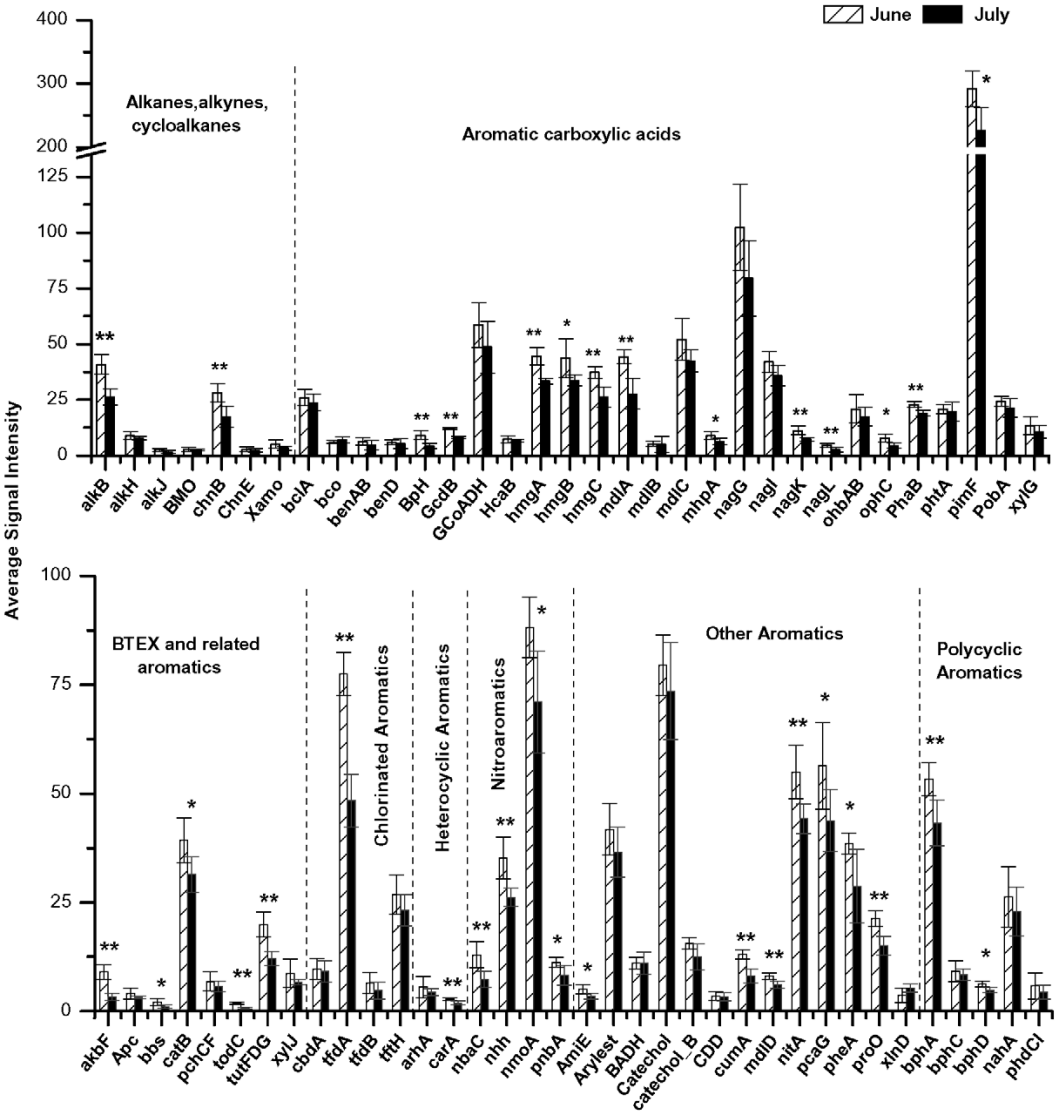
Hydrocarbon degradation genes in Marsh sediments. Average abundances of hydrocarbon degradation genes in Marsh sediments compared between June and July. Significant changes indicated by *for p<0.05 and **for p<0.01.

Macondo oil (MC252) collected from the wellhead was analyzed by GCMS (Figure 3a). The light crude consisted of *n*-alkanes nonane (C9) to triacontane (C30) with higher concentrations of the short-chain hydrocarbons (C9 to C16) and decreasing concentrations of long-chain hydrocarbons (C17 to C30). The pristane to phytane ratio of the crude oil was approximately 1.0, indicating minimal weathering of the oil [33], [34]. In contrast, the concentrations of *n*-alkanes in tar balls and sediments in July Marsh samples demonstrated a highly weathered signature with a pristane to phytane ratio of 0.21 (Figure 3b). Short-chain alkanes were undetected in tar balls and long-chain alkane concentrations steadily increased between C17 and C30.

### Bacterial Community Structure

Within salt marsh sediments, PhyloChip microarray-based analysis identified 12,018 distinct bacterial operational taxonomic units (OTUs) representing 77 phyla using the criteria in Hazen et al. (2010) (Data S1). The number of OTUs in the most abundant phyla is listed in Table 3 (see Table S1 for the complete list of detected phyla). Distinctive trends in the distribution of OTUs were observed during the 3-month time periods. The total number of OTUs in Inlet samples increased between June, July, and September, whereas the total number of OTUs in Marsh samples increased between June and July, but decreased in September. This same trend was observed within many of the individual phyla where *Firmicutes* and *Tenericutes* demonstrated the highest fold changes between June and September in Inlet samples increasing by approximately 8.5-fold in surficial samples (0–2 cm) and 3–3.5-fold in subsurface (8–10 cm) samples. The majority of dominant phyla in Marsh samples increased in number of OTUs in July but then decreased in September. *Bacteroidetes* increased by 5-fold between June and July but subsequently decreased by 9-fold in September. Other phyla, such as *Proteobacteria* and *Tenericutes* increased by 5- and 7-fold, respectively, in July, and decreased only by 3.6- and 1.2-fold in September. In contrast, *Firmicutes* did not display a decrease between July and September, but continued to increase in Marsh 0–2 cm sediments by 13-fold between June and September. Surficial sediments tended to have a higher number of OTUs than subsurface and Inlet and Marsh samples had comparable distribution of OTUs within the phyla.

Family-level identification of previously described hydrocarbon-degrading bacteria [35] (7082 OTUs) and their individual relative abundances (based on mean fluorescence intensities) in Inlet and Marsh sediments are summarized in Tables 4 and 5. Similar trends were observed in the relative abundance of bacteria between June and September as was seen with the relative richness (as determined by number of OTUs). Relative abundance tended to increase between June, July, and September in Inlet samples, however Marsh samples demonstrated an increase in July and a subsequent decrease in September. Exceptions to these trends included members of *Firmicutes* that increased in both Inlet and Marsh throughout the time period and *Actinobacteria* that decreased in September Inlet samples.

Non-metric multi-dimensional scaling (nMDS) analysis of the bacterial community based on the mean relative abundance of identified taxa (Figure 4) revealed three different groupings mainly associated by month: **Group I** (Inlet 0–2 cm June, Inlet 8–10 cm June, and Marsh 0–2 cm June), **Group II** (Marsh 8–10 cm June, Inlet 0–2 cm July, Inlet 8–10 cm July, Marsh 0–2 cm July, Marsh 8–10 cm July, and Inlet 0–2 cm September), and **Group III** (Inlet 8–10 cm September, Marsh 0–2 cm September, and Marsh 8–10 September). Analysis of similarities (ANOSIM) denoted significant (R = 1.0; p = 0.01) differences between the three groups. The overall relative bacterial abundances of these groups (Figure 5) indicated the taxa responsible for these significant differences. *Firmicutes* increased between Group I and Group III, with members of the classes *Bacilli* and *Clostridia* increasing in relative abundance by over 2-fold. *Tenericutes* and *Betaproteobacteria* additionally increased in relative abundance by ∼1.5-fold each. Phyla that decreased in relative abundance from Group I to Group III were *Verrucomicrobia* (2.3-fold), *Bacteroidetes* (2.3-fold), *Cyanobacteria* (1.8-fold), *Planctomycetes* (1.8-fold), *Gammaproteobacteria* (1.3-fold), and *Acidobacteria* (1.4-fold).

### Functional Gene Analysis

GeoChip microarray-based analysis identified 16,383 unique functional genes in 13 gene categories within salt marsh sediments (Data S2). Overall Inlet functional gene signal intensities showed moderate differences (R = 0.268; p<0.01) from functional genes detected in Marsh sediments between June and July 2010. The normalized signal intensity representing the relative abundance of all genes within specific categories in Inlet and Marsh samples were compared between June and July (Figure 6). Inlet functional genes within the categories of carbon cycling, metal resistance, organic remediation, and stress increased in July compared to June sediments, but were not significant (p>0.05). In contrast, Marsh sediment functional genes significantly decreased (p<0.05) between June and July in all functional gene categories, specifically carbon cycling, organic remediation, and stress.

Distinctive trends were observed in the average signal intensities of genes related specifically to hydrocarbon degradation in Inlet (Figure 7) and Marsh (Figure 8) sediments. Genes involved in hydrocarbon degradation increased in relative abundance between June and July in Inlet sediments but decreased in Marsh sediments. Genes known to degrade alkanes, cycloalkanes, aromatic carboxylic acids, chlorinated aromatics, polycyclic aromatics, and other aromatics decreased significantly (p<0.01 and p<0.05) in Marsh samples between June and July during the time oil concentrations were decreasing in Marsh sediments. In contrast, genes related to hydrocarbon degradation increased in signal intensities between June and July in Inlet sediments though not significantly (p>0.05).

## Discussion

Salt marshes ecosystems are highly productive and biologically rich environments that are sensitive to anthropogenic contamination such as oil spills [36], [37]. The overall impact to the system depends on the amount and type of oil, physical aspects of the marsh, and temperature. The *Deepwater Horizon* oil spill occurred approximately 77 km offshore during the late spring and by the time the oil reached the Alabama coast [6] in the early summer it presented primarily as weathered tar balls and/or mousse collecting within salt marsh sediments and along the shore. We detected oil in the Point Aux Pins salt marsh sediments in June 2010 that persisted through our samplings in September 2010. The amount of oil that affected the Alabama marsh was heterogeneously distributed and did not saturate the sediments as was observed in areas of coastal Louisiana [38]. Oil saturated sediments can form “pavements” that sink into the subsurface [39]. These reduced water-oil interfaces effectively preserve oil from microbial biodegradation and can remain sequestered for years. Analysis of sediment cores collected one year after the arrival of oil in the Point Aux Pins salt marsh detected no TPH within the top 30 cm of sediments (data not shown). The absence of “pavements” within this depth profile suggests that the heterogeneity and amount of oil in the sediment did not appear to favor oil preservation and may have been a key factor that contributed to the efficient removal of oil from these marsh sediments possibly by natural attenuation.

The relative distribution of the predominant bacterial phyla detected was approximately 7% *Bacteroidetes*; 11% *Actinobacteria*; 16% *Firmicutes;* and 46% *Proteobacteria* (Table 3). Dominance of *Proteobacteria* is consistent with the phylogenetic diversity observed in other estuarine salt marshes [43], [44], [45] and a Florida sandy beach impacted by the *Deepwater Horiz*on oil spill [23]. Both the relative abundance and number of OTUs of bacterial phyla increased during oiling, suggesting that not only did the relative community richness increase in response to the hydrocarbon input, but the relative intensities of those populations already at detectable levels responded as well. Members of hydrocarbon-degrading bacterial families that demonstrated the largest increases in relative abundance between June and July during the oiling of sediments were *Actinomycetaceae*, *Dietziaceae, Nocardioidaceae, Rhizobiaceae, Xanthobacteraceae, Erythrobacteraceae,* and *Aeromonadaceae.* Members of these families have been shown to degrade alkanes [46] and polynuclear aromatic hydrocarbons (PAHs) [47], [48], [49], [50]. Interestingly, several hydrocarbon-degraders detected in the salt marsh, such as *Aeromonadaceae, Bacillaceae, Pseudomonadales,* and *Shewanellaceae*, are also known to form biofilms and/or produce biosurfactants [51], [52], [53], [54], [55], [56] that can enhance oil degradation through gene transfer and oil emulsification [54], [57], [58].

Ordination of the bacterial community based on relative abundance revealed distinctive differences between groups of samples based primarily on month during the oil inundation (Figure 4). Increases in relative abundance of known hydrocarbon degraders, such as *Actinobacteria, Bacteroidetes,* and *Proteobacteria*, between June and July when oil concentrations were highest and subsequent decreases in September when concentrations were lower suggest a rapid microbial response to oil concentrations. In contrast, both *Bacilli* and *Clostridia* demonstrated significant increases in relative abundance in September. Classes of known hydrocarbon-degrading *Firmicutes* steadily increased in all samples between June and September even after oil was no longer detected in contrast to other hydrocarbon degraders that immediately declined when the oil was undetectable. Community enrichment of *Firmicutes* in salt marsh sediments has also been observed in response to hydrocarbon contamination in a simulated spill study [59] and may be used as an indicator of the later stages of hydrocarbon degradation when more recalcitrant compounds such as PAHs are present [23].

The function of the microbial community in salt marsh sediments also changed during the time of oiling. The overall microbial community functional structure was significantly (p<0.01) different between Marsh and Inlet sediments during the months of June and July when TPH concentrations were increasing in the Inlet and decreasing in the Marsh. While total functional gene relative abundances increased (12.7%) in Inlet samples between June and July in correlation with a 44% increase in total TPH, genes significantly (p<0.05) decreased by 25.2% in Marsh sediments where total TPH decreased by 36% in July compared to June (Figure 6; Table 2). This positive relationship between gene abundance, particularly hydrocarbon-degrading genes, and TPH suggests that community function was driven by oil concentrations. This association is further supported by an associated decrease in relative abundance of known hydrocarbon-degrading bacteria, specifically members of *Alteromonadaceae, Colwelliaceae,* and *Shewanellaceae,* between June and July in Marsh sediments. Significant (p<0.05) associations were observed between TPH and genes involved in degrading alkanes, alkynes, cycloalkanes, aromatic carboxylic acids, chlorinated aromatics, polycyclic aromatics, and other aromatics (Figure 8) suggesting that these genes were actively involved in degrading the oil that impacted the salt marsh. Similar findings have been reported in which hydrocarbon degrading genes were enriched in a deep-sea *Deepwater Horizon* oil plume [24], [27].

The salt marsh vegetation appeared to act as traps for the oil coming ashore. Oil was visible on marsh grass leaves and higher concentrations were detected within the Marsh sediments compared to the open Inlet during the early months of impact (Figure 2). Oil in the Marsh sediments, however, decreased significantly within one month and was no longer detected by September 2010, whereas oil in the Inlet sediments increased in July before declining in September. The greater decrease of oil in Marsh sediments compared to the Inlet sediments may have been a result of microbial degradation that was enhanced by the plant rhizosphere. The presence of marsh vegetation, such as *S. alterniflora* [40] and *J. roemerianus* [41], has been shown to decrease oil concentrations in sediments possibly due to plant uptake, increased aeration in the root zone, and/or production of root exudates that may promote oil mineralization and microbial hydrocarbon degradation. Enzymes, such as laccase, dehalogenase, nitroreductase, nitrilase, peroxidase, and cytochrome P450, supplied by roots and/or associated bacteria have been shown to aid in the detoxification of organic pollutants within the rhizosphere [e.g. 42,60,61]. Indeed, several of the genes that code for these enzymes (*phenol oxidase, rd, pbnA, ALN, nitA, lip, mnp, P450, BpH*) were significantly (p<0.05) higher in relative abundance in Marsh sediments compared to the Inlet suggesting a synergistic plant-microbe interaction within the salt marsh that may have been important in hydrocarbon degradation in these sediments.

Anaerobic degradation of hydrocarbons may also have contributed to the removal of oil from the salt marsh sediments. Regardless of oxygen-saturated overlying waters, dissolved oxygen has been shown to diffuse only within the top few millimeters of the sediment-water interface [62], [63], [64], thereby creating reducing conditions that may contribute to the anaerobic degradation of hydrocarbons in association with sulfate reduction, denitrification, and methanogenesis [65], [66], [67], [68]. Genes associated with anaerobic mechanisms, such as sulfate reduction (*dsrA/B*), methanogenesis (*mcrA*), and denitrification (*narG, nirK, nirS,* and *nosZ*), were detected throughout the salt marsh, along with several bacteria known to anaerobically degrade hydrocarbons, such as *Rhodocyclaceae, Geobacteraceae,* and *Desulfobacteraceae* (Table 5), suggesting that the anaerobic biodegradation of hydrocarbons could have occurred within the sediments.

Our results indicate that the microbial community structure and function of the coastal salt marsh was altered during the months of oil inundation from the *Deepwater Horizon* oil spill. The amount of incoming oil, its heterogeneous distribution within the sediments of the salt marsh, warm summer temperatures, and available nutrients may have played key roles in the efficient removal of oil that was undetectable within five months based on our samplings. The significant increase in response to the oil of the indigenous microbial population containing known hydrocarbon-degrading bacteria coupled with a subsequent increase in hydrocarbon degradation functional genes provides evidence that *in situ* bioremediation contributed to the degradation of oil at this site. These results suggest that in highly sensitive environments, such as salt marshes and estuaries that support a naturally occurring hydrocarbon-degrading microbial population, natural attenuation may be a preferable means of remediation to other mechanical strategies that can cause irreparable damage to the habitat. However, further comprehensive microbial studies are needed in Gulf of Mexico areas, where the threat of oil spills is an ongoing issue, in order to establish base-line microbial and ecological data so that the impacts of oil spills, such as the *Deepwater Horizon*, may be fully evaluated in the future.

## Supporting Information

Table S1 Total number of bacterial OTUs detected by PhyloChip in Inlet and Marsh sediments in all phyla.
(PDF)

Data S1 Total bacterial OTUs detected by PhyloChip for all samples. Positive fraction and normalized fluorescence intensity values are reported.
(XLSX)

Data S2 Functional gene normalized signal intensities detected by GeoChip.
(XLSX)
