# Correction: Microbial Community Analysis of a Coastal Salt Marsh Affected by the *Deepwater Horizon* Oil Spill

**DOI:** 10.1371/annotation/72b08ecf-1e78-4668-a094-c818def0e03f

**Published:** 2012-11-09

**Authors:** Melanie J. Beazley, Robert J. Martinez, Suja Rajan, Jessica Powell, Yvette M. Piceno, Lauren M. Tom, Gary L. Andersen, Terry C. Hazen, Joy D. Van Nostrand, Jizhong Zhou, Behzad Mortazavi, Patricia A. Sobecky

The Supporting Information files, Data S1 and Data S2, are corrupt. The correct files can be viewed here:

(Data S1)

(Data S2)

