# Supplementary material for: Microbial Community Analysis of a Coastal Salt Marsh Affected by the Deepwater Horizon Oil Spill
Source: PLoS One. 2012 Jul 18;7(7):e41305. doi: 10.1371/journal.pone.0041305 (PMC3399869; doi:10.1371/journal.pone.0041305)
Supplement: Table S1 — Total number of bacterial OTUs detected by PhyloChip in Inlet and Marsh sediments in all phyla. (PDF) [file pone.0041305.s001.pdf]

Table S1. Total number of bacterial OTUs detected by PhyloChip in Inlet and Marsh sediments in all phyla.

Phylum	Inlet 0-2 cm			Inlet 8-10 cm			Marsh 0-2 cm			Marsh 8-10 cm		
	**June**	**July**	**Sept**	**June**	**July**	**Sept**	**June**	**July**	**Sept**	**June**	**July**	**Sept**
ABY1_OD1	2	4	5	3	3	4	0	5	3	2	6	4
AC1	0	1	1	0	0	1	0	1	1	0	1	1
Acidobacteria	60	201	161	88	98	105	60	183	68	81	173	114
Actinobacteria	231	734	603	284	329	410	229	770	452	265	657	444
AD3	0	3	3	0	0	3	2	3	3	1	4	1
Aquificae	5	8	10	9	9	8	4	6	7	4	8	8
Armatimonadetes	1	13	8	2	2	6	2	11	3	1	15	5
Bacteroidetes	185	488	555	179	204	134	139	689	75	158	445	154
BH1	1	1	1	0	1	1	0	1	1	0	1	1
BRC1	0	4	3	1	2	2	1	3	1	3	4	3
Caldiserica	0	0	0	0	0	0	0	1	0	0	1	0
CCM11b	1	1	1	1	1	1	0	1	0	1	1	1
CD12	1	1	1	1	1	1	1	1	1	1	1	1
Chlamydiae	1	0	1	1	1	0	1	1	0	1	1	0
Chlorobi	5	15	17	11	12	12	3	17	9	8	19	12
Chloroflexi	46	139	99	68	71	79	61	158	73	73	169	100
Chrysiogenetes	0	1	0	0	0	0	0	1	1	0	1	0
Cyanobacteria	51	158	122	70	75	52	47	154	36	39	130	76
Deferribacteres	1	2	2	1	1	1	1	2	1	1	4	3
Dictyoglomi	1	2	1	1	1	1	0	2	1	1	2	1
Elusimicrobia	0	9	7	1	1	3	0	6	2	0	8	3
EM3	0	1	0	0	0	0	0	1	0	0	1	0
Fibrobacteres	1	8	8	2	1	3	1	9	2	1	6	3
Firmicutes	150	830	1262	276	312	975	117	1050	1560	157	1013	787
Bacilli	52	246	514	88	118	461	50	298	714	51	299	330
Clostridia	98	584	748	188	194	514	67	752	846	106	714	457
Fusobacteria	10	27	16	14	8	5	13	32	2	2	29	3
GAL15	1	1	2	1	1	1	0	1	1	1	1	2
Gemmatimonadetes	26	66	62	33	38	37	15	58	29	29	63	45
GN02	0	0	0	0	0	0	0	0	0	0	0	0
GN04	1	6	5	3	6	5	1	7	3	3	8	5
GN06	1	4	1	2	2	2	2	4	1	2	4	2
GN1	0	0	0	0	0	0	0	0	0	0	0	0
GN12	0	1	1	0	0	1	0	1	0	0	1	1
GOUTA4	0	0	0	0	0	0	0	0	0	0	0	0
HDBW-WB69	0	1	1	0	1	1	0	1	1	0	1	1
Hyd24-12	0	1	1	0	1	1	0	1	1	0	2	1
KSB1	2	3	2	4	2	2	0	3	2	3	2	2
LCP-89	0	5	5	4	4	4	1	2	1	5	4	5
LD1	1	2	0	1	1	0	1	2	1	1	2	0
Lentisphaerae	6	13	9	8	8	8	7	11	5	7	13	9
MD2896-B26	0	1	1	1	1	1	0	1	1	1	1	1
MSBL6	2	2	3	3	3	3	2	2	1	3	3	2
MVP-15	1	3	3	2	3	3	1	4	4	1	4	3
NC10	4	9	4	6	6	5	4	9	5	4	7	7
Nitrospirae	5	18	14	11	9	9	4	20	3	5	19	12
NKB19	2	10	9	6	7	5	2	11	3	5	11	8

Table S1. (continued)

Phylum	Inlet 0-2 cm			Inlet 8-10 cm			Marsh 0-2 cm			Marsh 8-10 cm		
	June	July	Sept	June	July	Sept	June	July	Sept	June	July	Sept
OP1	0	1	0	0	0	0	0	1	0	0	1	1
OP11	1	1	2	1	3	2	1	2	2	2	2	2
OP3	4	8	8	6	7	7	4	7	5	4	8	6
OP8	3	6	8	3	5	5	3	6	1	6	9	8
OP9	3	6	7	3	3	3	2	6	4	4	10	6
PAUC34f	1	3	2	1	1	1	1	2	1	1	2	1
Planctomycetes	76	166	117	90	85	79	74	157	45	77	147	97
Proteobacteria	1140	3196	3843	1441	1560	1719	713	3625	1019	1084	2439	1684
Alphaproteobacteria	151	720	820	154	138	332	113	903	206	108	457	312
Betaproteobacteria	145	411	487	161	182	278	109	452	249	124	320	261
Deltaproteobacteria	164	560	438	244	270	278	159	595	172	209	567	318
Epsilonproteobacteria	36	54	107	88	98	89	44	90	27	84	104	90
Gammaproteobacteria	642	1446	1983	792	869	740	288	1577	364	558	986	699
Unclassified *Proteobacteria*	0	1	4	0	1	0	0	3	0	0	1	1
Zetaproteobacteria	2	4	4	2	2	2	0	5	1	1	4	3
SAR406	4	26	20	12	13	17	5	25	15	10	24	19
SBR1093	0	1	4	1	0	1	1	4	1	2	4	1
SC4	2	3	3	2	2	3	0	3	2	2	3	3
SM2F11	0	0	0	0	0	0	0	0	0	0	0	0
SPAM	3	4	6	2	2	5	2	6	5	3	6	5
Spirochaetes	16	67	64	31	36	44	19	65	35	20	73	43
SR1	2	4	3	3	4	4	2	4	4	2	4	4
Synergistetes	3	10	11	4	6	6	3	11	6	2	11	7
Tenericutes	8	59	68	18	19	53	12	81	68	17	72	39
TG3	0	5	6	3	3	4	1	4	1	4	6	4
Thermi	3	10	9	4	4	8	4	8	9	4	10	11
Thermodesulfobacteria	1	4	3	3	2	3	1	3	2	2	4	4
Thermotogae	0	1	2	1	0	0	0	4	0	0	3	2
TK-SH1	0	0	1	0	0	0	0	0	0	0	0	0
TM6	3	9	5	4	4	3	3	9	4	4	13	6
TM7	1	3	1	1	1	1	1	3	0	1	3	1
Unclassified	0	3	2	1	1	2	0	3	2	1	3	2
Verrucomicrobia	36	142	99	35	37	37	34	138	22	26	90	41
VHS-B3-43	0	0	0	1	1	0	0	0	0	1	1	1
WPS-2	1	3	3	2	1	2	0	3	1	0	2	3
WS1	0	1	2	1	1	1	0	1	2	0	2	2
WS2	1	1	1	1	1	1	1	1	1	1	1	1
WS3	15	36	28	23	23	25	15	33	17	21	34	23
ZB2	0	0	0	0	0	0	0	0	0	0	0	0
ZB3	1	1	1	1	1	1	1	2	0	1	2	1
**Total**	**2133**	**6577**	**7339**	**2797**	**3052**	**3932**	**1625**	**7463**	**3638**	**2172**	**5835**	**3859**
